# Teaching cards as low-cost and brief materials for teaching basic life support to 6–10-year-old primary school children – a quasi-experimental combination design study

**DOI:** 10.1186/s12887-022-03730-3

**Published:** 2022-11-08

**Authors:** Anita Kovács, Henrietta Bánfai-Csonka, József Betlehem, Luca Anna Ferkai, Krisztina Deutsch, János Musch, Bálint Bánfai

**Affiliations:** 1 Institute of Emergency Care and Pedagogy of Health, University of Pécs Faculty of Health Sciences, Vörösmarty Street 4, 7621 Pécs, Hungary; 2 Present Address: Department of Emergency Medicine, University of Pécs, Clinical Centre, Ifjúság Street 13, 7624 Pécs, Hungary

**Keywords:** Basic Life Support, KIDS SAVE LIVES, Resuscitation training, Schoolchildren, Teaching cards, Attitude, Teaching, Education

## Abstract

**Aim:**

Teaching Basic Life Support (BLS) in schools is a key initiative to improve the survival rates after out-of-hospital cardiac arrest. Low-cost training materials can reach a wider population. Our aim was to compare the effectiveness of using teaching cards with the traditional instructor-led and combined methods on BLS skills and attitude and to evaluate the long-term effects after two months.

**Methods:**

A quasi-experimental combination design study. Two hundred sixty-three schoolchildren aged 6 to 10 years were assigned to three groups with different methods to teach BLS: teaching card group (*n* = 100), traditional instructor-led teaching group (*n* = 91), combined teaching group (*n* = 72). BLS skills and attitude were measured and compared before the training (T0), after the training (T1), and two months later (T2).

**Results:**

BLS skills improved in every group at T1 compared to T0 (*p* < 0.001) and remained higher at T2 than at T0 in almost all cases (*p* < 0.001). Skill performance was similar in most of the skills between the three groups at T1. The best skill scores acquired were calling the ambulance and the correct hand position by chest compression. Positioning the head during check the breathing was more effective in the traditional group (48.4%) and combined group (61.1%) than in the teaching card group (19.0%) (*p* < 0.001) at T1. However, some skills improved significantly in the teaching card group at T2: check breathing for 10 s (*p* = 0.016); positioning the head by check breathing (*p* < 0.001); and positioning the head by ventilation (*p* = 0.011). Attitude did not change significantly in any of the groups (*p* > 0.05). Furthermore, the level of attitude was inferior in the teaching card group compared with the traditional (*p* = 0.005), and the combined groups (*p* = 0.049).

**Conclusion:**

Using low-cost materials for teaching BLS for young schoolchildren can improve their skills, however, could not improve attitudes. Teaching cards were not inferior compared to traditional and combined methods in some skills but inferior in others. Therefore, hands-on training opportunity is still important. Teaching cards are useful for long-term learning. To learn correctly the whole sequence of BLS is difficult for 6 to 10 years-old children, however, they are able to learn more BLS-related skills separately.

## Introduction

Out-of-hospital cardiac arrest (OHCA) is a leading cause of death worldwide [1]. Quick and effective bystander help can save lives. Early high-quality cardiopulmonary resuscitation (CPR) and automated external defibrillator (AED) use improves the chance of survival with better neurological outcomes [2, 3]. However, the willingness to help is lower than expected [4, 5]. The European Resuscitation Council (ERC) stated that educational efficiency is one of the three multiplicands that would affect survival [6]. Therefore, increasing awareness, willingness to perform first aid and CPR, and participation in training is a key issue by leading health care authorities [7, 8].

Introducing CPR training at schools is one of the key initiatives to improve the community response after OHCA. The KIDS SAVE LIVES campaign has been successfully implemented worldwide [9–11]. A wide range of people is able to teach schoolchildren for CPR successfully (e.g. healthcare professionals, trained teachers, healthcare students, etc.) [12, 13]. However, there are several limitations of the implementation of first aid and CPR education in schools (e.g. lack of time, lack of knowledge, financial reasons, lack of instructor, etc.) [14–16].

To increase the knowledge and skills related to Basic Life Support (BLS) many types of training are available. The most common type is the instructor-led training, however, it has some disadvantages (e.g. no time for participation, not enough time to practice on manikins, inconsistency in instructions, inconvenience, anxiety in an unfamiliar setting) [17, 18]. Therefore, self-instruction courses and new alternative methods can be promising which enables mass training capacity gets improved. Usually, these programs include videos, virtual reality (VR), smartphone apps, or personal manikins with or without any feedback possibility. Comparing such training methods with traditional training can lead to good quality knowledge scores, but poorer skill performance [19]. Another challenge can be the availability of these alternative methods (e.g. due to financial reasons). New, repeatable, low-cost training methods include the potential to reach a much larger target population.

Children learn different way than adults so teaching materials should created to childhood specialities [20]. Several prior studies focused to measure the effectiveness of CPR training in different age groups, however, the best way to teach BLS to children is still unknown [21–24].

Our aim was to compare the efficacy of our self-made BLS „teaching cards” with the traditional, instructor-led, and combined teaching on skills and attitude of 6–10-year-old schoolchildren. We hypothesized that using teaching cards is non-inferior to the traditional and the combined teaching (neither in improving skills nor in attitude).

## Methods

A quasi-experimental combination design study was conducted to investigate and compare the effectiveness of BLS teaching cards, the traditional instructor-led and the combined teaching among 6-to-10-year-old primary school children.

### Participants

Schoolchildren from the first four classes of the primary school (6–10-year-old) were participated in this study. Children in this study were from three urban primary schools located in Kecskemét, Hungary. The study was conducted between December 2019 and March 2020. All students were involved who attended the given classes of the schools. Children who had not the parental consent were excluded from the study.

### Study design

Participants were randomly assigned to three different groups: teaching card group, traditional teaching group, and combined teaching group. Randomization was based on the three different schools to avoid contaminating the different methods in the same school. The content of the training was the sequence of BLS followed the ERC 2015 guidelines [25].

In the traditional teaching group, participants went through a traditional training using a standard BLS manikin (AMBU® Man W model; AMBU A/S, Baltorpbakken 13, DK 2750 Ballerup, Denmark) by an experienced instructor. The training included a 45 min long session (15 min theoretical background and 30 min hands-on training).

**Fig. 1:**
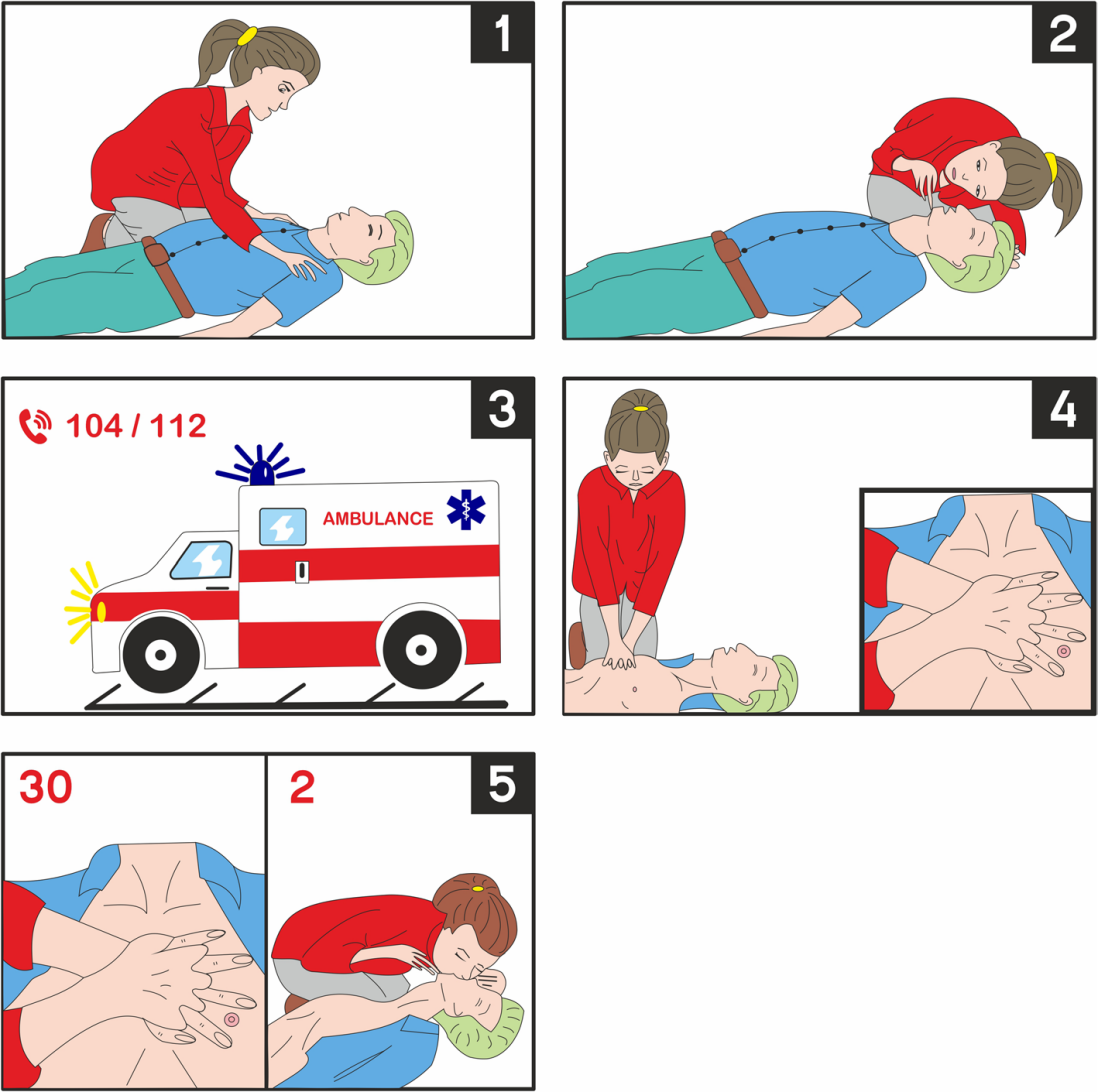
Teaching-cards about adult Basic Life Support (BLS). 1 = Make sure that you, the victim (or anybody) are safe. Shake the victim by the shoulders and ask: Hello, are you all right?. 2 = Check the breathing: (a) tilt the patient’s head back; (b) use the look-listen-feel formula (looking at the chest movements, listening, and feeling the air-flow); (c) check the breathing for 10 s. 3 = Call the ambulance at 112. 4 = Start CPR: a Kneel one side of the victim; b Place the heel of the hand in the center of the chest (the lower third of the sternum); c Place the heel of your other hand on top of the first hand and interlock your fingers; d Keep your arms straight; e Position yourself vertically above the victim’s chest and press down on the sternum at least 5 cm (but no more than 6 cm); f After each compression, release all the pressure on the chest without losing contact between your hands and the chest of the victim; g Repeat at a rate of 100–120 min.−1. 5 = Continue CPR: a Repeat chest compressions 30 times; b Tilt the head back; c Blow the air into the patient’s mouth or nose two times (and keep the other closed); d Continue with the ratio of chest compression and ventilation of 30:2

In the teaching card group, children get the appropriate theoretical background about the BLS and an explanation of how to use the teaching cards. This group was led by an experienced instructor, as well. Teaching cards contained pictures (first page) and a short subscription (opposite page) about all elements of BLS (Fig. 1). The hands-on training was not part of this group. The training lasted 20 min (15 min theoretical background and 5 min about the usage of the teaching cards).

Teaching card descriptions (1–5) are based on the ERC 2015 BLS Guidelines [25].

In the combined teaching group the two prior methods were combined: traditional instructor-led training expanded with the teaching cards. The training included a 45 min long session (15 min theoretical background and 30 min hands-on training, and additional instructions were given about the usage of teaching cards).

The instructor in our study was a paramedic with ERC related instructor license and with more years of experience in teaching BLS to schoolchildren. The instructor was the same person in every group to avoid possible biases based on personal differences.

Teaching cards in the teaching card group and the combined teaching group were possible to use not only during the training session but also after it (except in the tests). The ratio of instructor and children was 1:10 in the traditional and combined teaching groups.

All children’s BLS skills and attitudes were tested prior to the educational session (T0). To evaluate skills, a scenario with a manikin was presented with the following instruction: „A person is lying on the ground with a health emergency and needing help. Please show what would you do to help the victim?” Childrens’ performance was assessed using a checklist by an independent BLS instructor blinded the study groups. Ten independent elements of BLS (check safety, check response, check breathing for 10 s by positioning the head and using look-listen-feel, call the ambulance, hand position of chest compression, correct number of chest compressions, positioning the head for ventilation, the correct number of ventilation) were assessed by an experienced instructor. For evaluating BLS skills together (the whole sequence), the proportion of students who performed successfully all of the elements, and/or passed a minimum of 60% of the elements [26]. For scoring, 0 points was given in case of missing or incorrect elements and 1 point was given if the skills were correct.

The attitude was evaluated by answering the following question: „A person is lying on the ground with a health emergency and needing help. Would you help him/her?” The question was asked orally and answers (yes/no) were documented on the checklist by the instructor before the skill assessment at T0, T1 and T2.

After the educational session, children were assessed within one week (T1) and two months later (T2). Post-training measurements were carried out with the same methods described by T0.

### Sample size calculation

The sample size calculation was made by G*Power 3.1 software (Heinrich-Heine-Universität, Düsseldorf, Germany) with an effect size of 0.3, an alpha-error probability of 0.05, and a statistical power of 0.95 with a degree of freedom of 2. It was determined that 172 children were required. We tried to recruit as many students as possible from the first four classes of the three primary schools.

### Statistical analysis

Statistical analysis was conducted using SPSS 24.0 (Statistics Package for Social Sciences, Chicago, IL, USA). Descriptive statistics were performed (percentage, mean, standard deviation) to describe the sample. Categorical variables (absolute and relative frequencies) were compared by using McNemar-test (pairwise intragroup comparison) and Chi-square test or Fisher’s-exact test as appropriate (intergroup comparison). Similarly as described by Abelairas-Gómez et al. [20], the effect size was calculated, as well. Cohen’s g was used for intragroup comparisons (small: 0.05–0.15; medium: 0.15–0.25; large ≥ 0.25). Cramer’s V was used for intergroup analysis (small: 0.10–0.30; medium: 0.30–0.50; large ≥ 0.50). One-way ANOVA was applied to compare the age of the different groups. A p-value less than 0.05 was considered to be statistically significant.

### Ethics approval and consent to participate

The participants, their parents, and teachers received written and oral information prior to study commencement. They were informed of their right to withdraw at any time with no personal consequences. The children’s parents gave written informed consent before their child entered the study. The study protocol was approved by the Regional and Institutional Research Ethical Committee of the Clinical Centre of the University of Pécs (approval number: 7756 – PTE 2019).

## Results

**Fig. 2:**
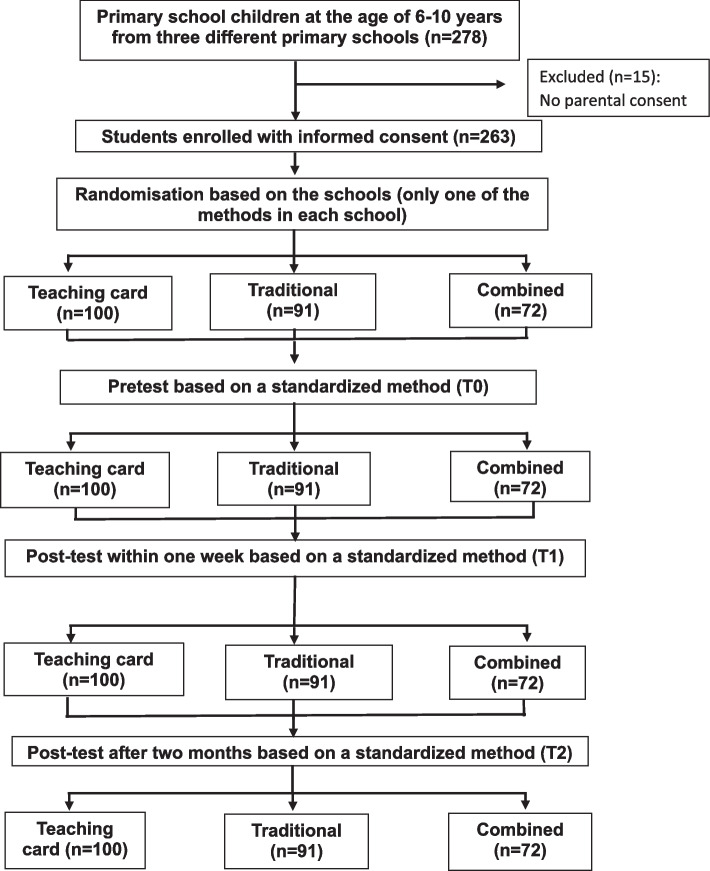
Study flow chart

A total of 278 primary school students were recruited for the study. Fifteen of the participants were excluded because of the exclusion criteria (students had not the parental consent. As such, 263 students were allocated to the training groups: 100 (38.0%) to the teaching card group, 91 (34.6%) to the traditional teaching group, and 72 (27.4%) to the combined teaching group (Fig. 2).

Gender, age, and class distribution were similar between the three groups. The main characteristics of the participants are shown in Table 1. Participants did not participate in any formal BLS training before the study.

**Table 1:** The main characteristics of the children

Characteristics	In total (*N* = 263)	Teaching card (*n* = 100)	Traditional (*n* = 91)	Combined (*n* = 72)	*p*-value
Boys	133 (50.6)	51 (51)	47 (51.6)	35 (48.6)	0.923
Age (mean ± SD)	8.49 ± 1.1	8.33 ± 1.2	8.51 ± 1.1	8.62 ± 0.8	0.07
Class					0.430
First	67 (25.5)	25 (25.0)	29 (31.9)	13 (18.1)	0.143
Second	70 (26.6)	31 (31.0)	22 (24.2)	17 (23.6)	0.670
Third	67 (25.5)	23 (23.0)	28 (30.8)	16 (22.2)	0.587
Forth	59 (22.4)	21 (21.0)	12 (13.2)	26 (36.1)	0.08

Data are presented as numbers and proportions in brackets (except age)

**Fig. 3:**
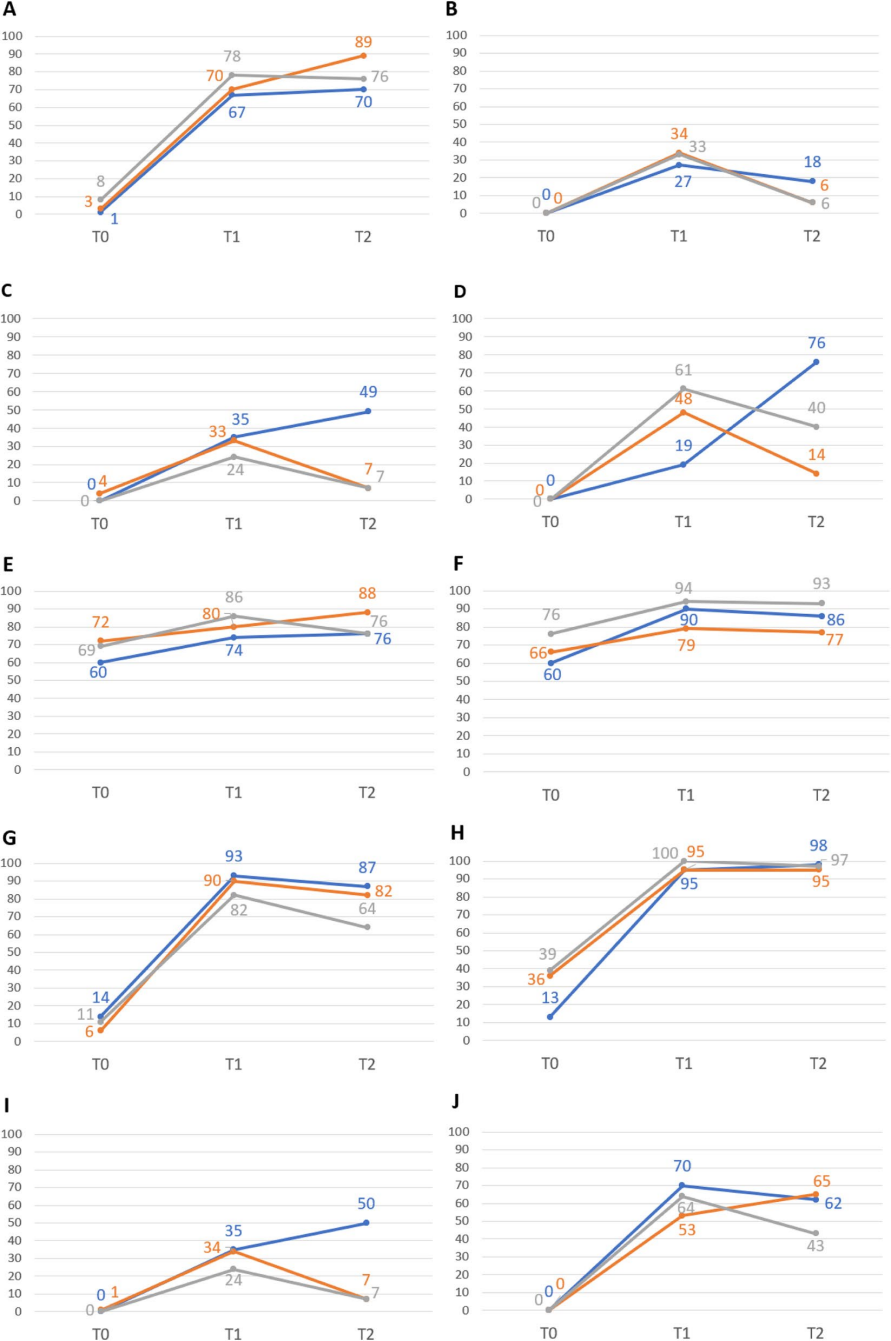
Effectiveness ratio (%) of different BLS skills evaluated individually. T0 = pretest before the training; T1 = post test within one week; T2 = post-test after two months; BLS = Basic Life Support; A = check safety; B = check consciousness; C = check breathing (10 s); D = check breathing (positioning the head); E = check breathing (look-listen-feel); F = call the ambulance (112); G = chest compression (30); H = chest compression (hand position); I = ventilation (positioning the head); J = ventilation (2 times); blue colour = teaching card group, orange colour = traditional group, grey colour = combined group

**Fig. 4:**
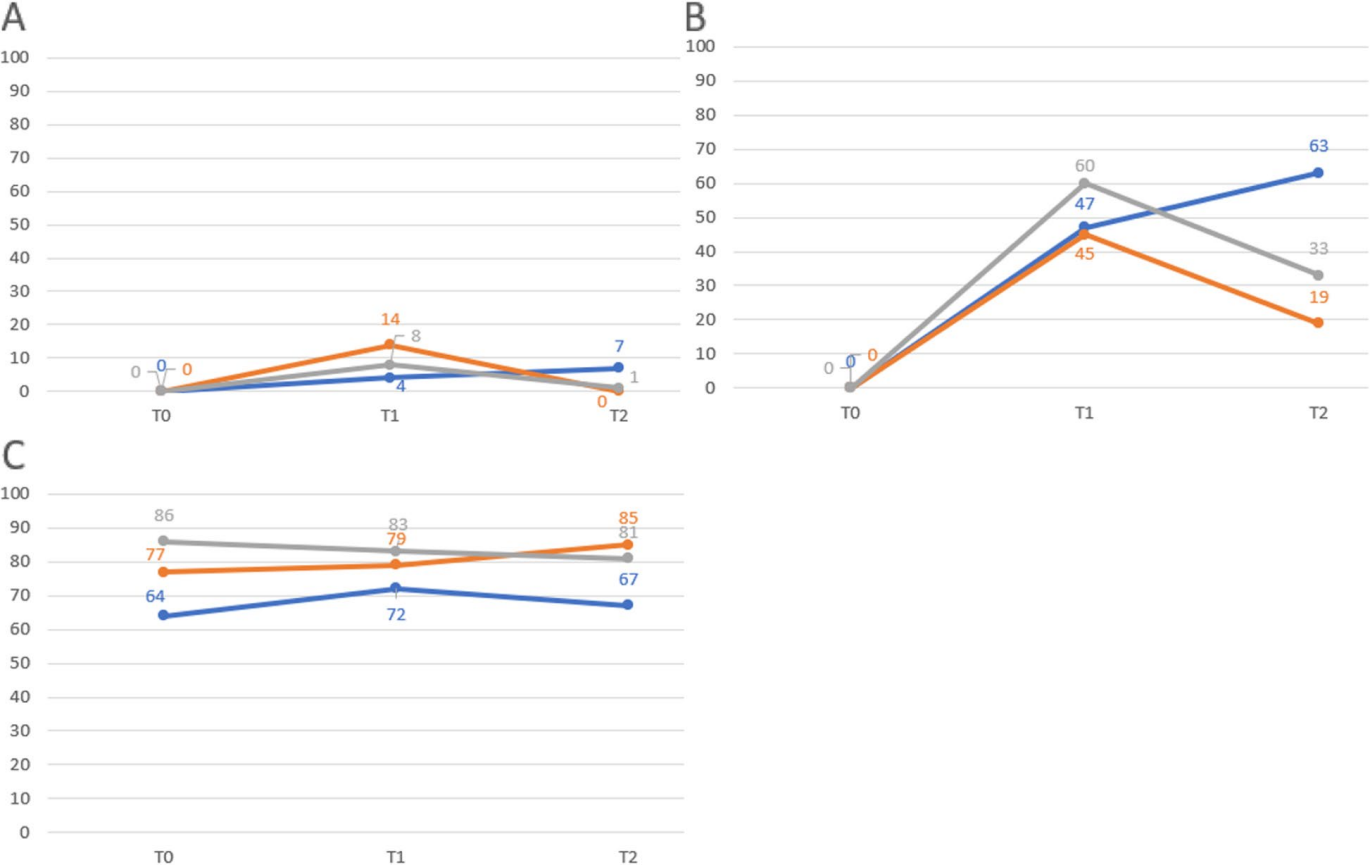
Effectiveness ratio (%) of different BLS skills in total and the attitude of the schoolchildren. T0 = pretest before the training; T1 = post test within one week; T2 = post-test after two months; BLS = Basic Life Support; A = total BLS score (every steps were correct); B = total BLS score ((≥ 60% was correct); C = Ratio of „yes” answers to the question: „A person is lying on the ground with a health emergency and needing help. Would you help him/her?”; blue colour = teaching card group, orange colour = traditional group, grey colour = combined group

BLS performance improved in every group after the training (from T0 to T1; *p* < 0.001 in every case (Tables 2 and 3, Figs. 3 and 4).

**Table 2:** Results of intragroup comparisons

Skills	*p*-values (effect sizes)								
	Teaching card			Traditional			Combined		
	T0 vs. T1	T0 vs. T2	T1 vs. T2	T0 vs. T1	T0 vs. T2	T1 vs. T2	T0 vs. T1	T0 vs. T2	T1 vs. T2
Check safety	< 0.001 (0.20)	< 0.001 (0.22)	0.648 (0.16)	< 0.001 (0.22)	< 0.001 (0.20)	< 0.001 (0.22)	< 0.001 (0.18)	< 0.001 (0.24)	0.546 (0.14)
Check consciousness	-^*^	-^*^	0.122 (0.16)	-^*^	-	< 0.001 (0.20)	-^*^	-^*^	< 0.001 (0.22)
Check breathing (10 s)	-^*^	-^*^	0.016 (0.20)	< 0.001 (0.24)	0.625 (0.14)	< 0.001 (0.20)	-^*^	-^*^	< 0.001 (0.20)
Check breathing (positioning the head)	-^*^	-^*^	< 0.001 (0.22)	-^*^	-	< 0.001 (0.22)	-^*^	-^*^	< 0.001 (0.20)
Check breathing (look-listen-feel)	< 0.001 (0.24)	0.110 (0.18)	0.834 (0.14)	< 0.001 (0.24)	< 0.001 (0.20)	< 0.001 (0.20)	< 0.001 (0.22)	0.072 (0.18)	0.012 (0.20)
Call the ambulance (112)	< 0.001 (0.20)	< 0.001 (0.17)	0.454 (0.14)	< 0.001 (0.20)	< 0.001 (0.23)	0.824 (0.14)	< 0.001 (0.22)	< 0.001 (0.19)	1 (0.16)
Chest compression (30)	-^*^	-^*^	0.238 (0.14)	< 0.001 (0.20)	< 0.001 (0.20)	0.143 (0.18)	< 0.001 (0.22)	< 0.001 (0.22)	< 0.001 (0.20)
Chest compression (hand position)	-^*^	-^*^	0.453 (0.20)	< 0.001 (0.16)	< 0.001 (0.18)	1 (0.20)	< 0.001 (0.24)	< 0.001 (0.22)	1 (0.12)
Ventilation (positioning the head)	-^*^	-^*^	0.011 (0.18)	< 0.001 (0.20)	0.765 (0.17)	< 0.001 (0.20)	-^*^	-^*^	< 0.001 (0.23)
Ventilation (2 times)	-^*^	-^*^	0.243	-^*^	-^*^	< 0.001 (0.23)	-^*^	-^*^	< 0.001 (0.21)
Total BLS score (every step were correct)	-^*^	-^*^	0.213 (0.15)	-^*^	-^*^	-^*^	-^*^	-^*^	0.435 (0.19)
Total BLS score (≥ 60% were correct)	-^*^	-^*^	0.011 (0.21)	-^*^	-^*^	< 0.001 (0.24)	-^*^	-^*^	0.001 (0.23)
Attitude									
A person is lying on the ground with a health emergency and needing help. Could you help him/her?	0.139 (0.15)	0.690 (0.13)	0.424 (0.20)	0.839 (0.18)	0.210 (0.21)	0.405 (0.15)	0.791 (0.18)	0.424 (0.16)	0.774 (0.20)

^*^Statistical analysis was not appropriate because the number of correct skills was 0 at these time points

*P*-values are added and effect sizes in brackets. Intragroup effect sizes were small or medium (0.05–0.25) in every case

*T0* Pretest before the training, *T1* Post test within one week, *T2* Post test after two months, *BLS* Basic Life Support

**Table 3:** Results of intergroup comparisons

Skills	*p*-values (effect sizes)								
	T0			T1			T2		
	TCG vs. TG	TCG vs. CG	TG vs. CG	TCG vs. TG	TCG vs. CG	TG vs. CG	TCG vs. TG	TCG vs. CG	TG vs. CG
Check safety	0.268 (0.24)	0.016 (0.31)	0.162 (0.32)	0.621 (0.23)	0.122 (0.32)	0.284 (0.34)	0.025 (0.40)	0.354 (0.31)	0.031 (0.38)
Check consciousness	-^*^	-^*^	-^*^	0.289 (0.26)	0.370 (0.32)	0.922 (0.30)	0.289 (0.31)	0.016 (0.40)	0.986 (0.24)
Check breathing (10 s)	-^*^	-^*^	-^*^	0.767 (0.23)	0.109 (0.35)	0.190 (0.32)	0.767 (0.29)	< 0.001 (0.42)	0.929 (0.27)
Check breathing (positioning the head)	-^*^	-^*^	-^*^	< 0.001 (0.40)	< 0.001 (0.38)	0.105 (0.24)	< 0.001 (0.41	< 0.001 (0.38)	< 0.001 (0.44)
Check breathing (look-listen-feel)	0.136 (0.35)	0.081 (0.28)	0.146 (0.34)	0.855 (0.30)	0.109 (0.34)	0.082 (0.34)	0.855 (0.31)	< 0.001 (0.40)	0.929 (0.31)
Call the ambulance (112)	0.397 (0.29)	0.024 (0.36)	0.189 (0.32)	0.036 (0.31)	0.293 (0.28)	0.005 (0.41)	0.036 (0.45)	0.145 (0.29)	0.005 (0.33)
Chest compression (30)	0.06 (0.36)	0.576 (0.29)	0.731 (0.28)	0.471 (0.29)	0.026 (0.35)	0.130 (0.30)	0.471 (0.24)	< 0.001 (0.40)	0.007 (0.37)
Chest compression (hand position)	< 0.001 (0.35)	< 0.001 (0.34)	0.372 (0.31)	0.878 (0.29)	0.054 (0.33)	0.630 (0.28)	0.878 (0.27)	0.738 (0.27)	0.396 (0.32)
Ventilation (positioning the head)	-^*^	-^*^	-^*^	0.840 (0.30)	0.399 (0.31)	0.153 (0.31)	0.014 (0.35)	0.014 (0.34)	0.706 (0.30)
Ventilation (2 times)	-^*^	-^*^	-^*^	0.892 (0.25)	0.109 (0.26)	0.146 (0.36)	0.892 (0.28)	< 0.001 (0.36)	0.929 (0.24)
Total BLS score (every step were correct)	-^*^	-^*^	-^*^	0.018 (0.35)	0.086 (0.35)	0.003 (0.35)	< 0.001 (0.40)	< 0.001 (0.40)	0.066 (0.35)
Total BLS score (≥ 60% were correct)	-^*^	-^*^	-^*^	0.788 (0.28)	0.099 (0.30)	0.063 (0.35)	< 0.001 (0.41)	< 0.001 (0.35)	0.045 (0.30)
Attitude									
A person is lying on the ground with a health emergency and needing help. Would you help him/her?	0.051 (0.29)	0.001 (0.37)	0.294 (0.29)	0.254 (0.33)	0.083 (0.31)	0.705 (0.28)	0.005 (0.33)	0.049 (0.29)	0.714 (0.28)

^*^Statistical analysis was not appropriate because the number of correct skills was 0 at these time points

*P*-values are added and effect sizes in brackets. Intergroup effect sizes were small or medium (0.10–0.50) in every case

*T0* Pretest before the training, *T1* Post test within one week, *T2* Post test after two months, *TCG* Teaching card group, *TG* Traditional group, *CG* Combined group, *BLS* Basic Life Support

The baseline skills were similar in the traditional (TG) and combined (CG) teaching groups but were superior in some skills than in the teaching card (TCG) group: check safety (TCG vs. CG, *p* = 0.016); call the ambulance (TCG vs. CG, *p* = 0.024); hand position in chest compression (TCG vs. TG and CG (*p* < 0.001 in both cases). Skill performance was similar in most of the cases between the three groups after the training (at T1). The best skill scores acquired were calling the ambulance and the correct hand position by chest compression in every group. However, positioning the head during check the breathing was more effective in TG (48.4%) and CG (61.1%) than in TCG (19.0%) (*p* < 0.001 in both cases). Calling the ambulance was more successful in groups using cards (TCG: 90%, CG: 94.4%) than in the TG (79.1%) (*p* < 0.05 between TCG and CG vs. TG, in both cases). In addition, the number of chest compressions was correct in TCG in a higher proportion than in CG (93.0% vs. 81.9%, *p* = 0.026).

In the majority of the cases, the performance in TCG was superior to TG and CG two months after the training (Tables 2 and 3, Figs. 3 and 4). Some skills improved significantly in TCG at T2: check breathing for 10 s (*p* = 0.016); positioning the head by check breathing (*p* < 0.001); and positioning the head by ventilation (*p* = 0.011).

Assessing the results together, the total BLS sequence was performed correctly and in the appropriate order by only an extreme minority of young children after the training (at T1): 4.0% of TCG, 14.3% of TG, and 8.3% of CG. TCG had the highest proportion performing the whole sequence correctly (7.0%) compared to TG (0%) and CG (1.4%); *p* < 0.05 in both cases at T2. If the pass rate is defined by 60% (at least 60% of the BLS sequence is correct), group members performed similarly at T1 (TCG: 47.0%, TG: 45.1%, and CG: 59.7%). Effectiveness declined significantly in TG and CG (from 45.1% to 18.7% in TG, *p* < 0.001; from 59.7% to 33.3% in CG, *p* = 0.001) at T2, however, improved in TCG (from 47.0% to 63:0%, *p* = 0.011). Summarized data about the performance are shown in Tables 2, 3 and Figs. 3, 4. Attitude did not change significantly in any of the groups over time (from T0 to T1 and T2). However, the intergroup comparison showed differences between the level of attitude at T2: the lowest level of attitude was visible in TCG (67% compared to 84.6% in TG and 80.6% in CG; *p* < 0.05 in both cases) (Tables 2 and 3, Fig. 4).

## Discussion

In this quasi-experimental combination design study teaching cards were analyzed as an alternative low-cost method and a comparison was made between this new method and the traditional and the combined teaching among young (6-to-10-year-old) primary schoolchildren. The results showed that the teaching card teaching was non-inferior in the majority of the cases compared with the other teaching methods, however, it was inferior in other cases (e.g. check breathing). Some skills were the most effective after the traditional teaching but skill performance improved in every group after the training. Interestingly, the best skill performance was measured in the teaching card group after two months. Based on the results, every type of training were able to improve skills, however, learning some skills effectively requires a formal practical session. Teaching cards were able to introduce the importance of BLS and to learn some parts of it (e.g. call the ambulance). On the other hand, using teaching cards alone was not able to learn and do the whole sequence of BLS correctly. Attitude did not change in any of the groups over time. Furthermore, the attitude related to starting BLS was the lowest in the teaching card group. Based on this latter point, using teaching cards was not able to change the attitude in our study.

Implementation of BLS into the school curricula is a key initiative to improve knowledge, skills, and attitude which improves the rates of bystander CPR and survival rates [7, 8]. KIDS SAVE LIVES project suggests starting learning CPR at the age of 12 [9, 27], however, education can be useful for younger children, as well [6]. In general, younger children are not able to perform effective chest compressions [21, 22, 28, 29]. However, they are able to learn the main components of BLS which may be an effective sensitization [24, 30]. Although the opinions are positive by children and teachers implementation is still challenging [16, 21]. Major difficulties are the lack of instructor, time, and equipment [14, 15]. A wide range of people is able to teach schoolchildren for CPR successfully (e.g. healthcare professionals, trained teachers, healthcare students, etc.) [12]. ERC recommends that one of the best ways is if CPR training is held by the own schoolteachers of the children after the appropriate training [13]. In our study, the instructor was an experienced paramedic. The reason why a paramedic was the instructor in our study is that only a few schoolteachers with the appropriate experience and knowledge are available in Hungary at this time. Unfortunately, CPR training is not part of the university curricula of schoolteachers yet. However, further studies would be required and useful to evaluate the effectiveness of different BLS teaching methods teached by schoolteachers.

Children learn in different ways than adults thus educational materials fitting to the appropriate age groups are essential [22, 31]. As a result of advances in technology, different alternative methods are available in BLS teaching (e.g. video-based method, VR technology, smartphone apps) [19, 26, 32, 33]. These methods are useful but require specific technological resources (software and/or apps connecting to a manikin) which may be an obstacle in implementation in settings with a lower level of economic status. In addition, some children are too young to use these devices, and/or the schools do have not the appropriate resources. To address this problem, new, low-cost, and „easy-to-use” educational materials adapted to the age may be useful [11, 20].

A recent study’s results showed that new didactical tools are more effective among young children than the traditional method [20]. In our present study, using teaching cards showed that such materials can improve some skills successfully, however, had no effects on the helping attitude. Every skills improved after training, and some of them improved further over time (after two months), as well. The reason for this can be that teaching cards are repeatable and easily accessible sources that promote continuous learning opportunities. Visual support by the teaching cards was useful to memorize some elements of the BLS sequence (e.g. the correct emergency phone number) and to remember the correct skills after two months. Interestingly, children in the combined group were less effective compared to the teaching card (despite using also teaching cards) and the traditional groups after two months. This difference may be between the teaching card and combined groups due to the higher level of self-confidence in the combined group because of the hands-on training possibility (they did not feel the necessity of teaching cards to repeat the BLS sequence continuously). Furthermore, in some skills (e.g. positioning the head by checking the breathing) the traditional method was the most successful which indicates the need for hands-on training. In some cases, children were able to remember the appropriate element, but they were not able to perform it correctly (e.g. check the breathing with the appropriate method but it took only two or three seconds, or consciousness was checked only by asking the victim without shaking his/her shoulders, etc.).

In prior studies, self-instruction kits were able to improve the knowledge and the skills of participants so these can be an alternative to traditional methods [34]. Based on our results, an optimal mixture of traditional and alternative methods reflecting the appropriate age group can be useful to improve BLS skills. To involve a larger population, self-instruction kits can be beneficial in some factors but hands-on training opportunities and real-time feedback by an instructor are essential for participants. However, the first links of the chain of survival (recognition and calling the ambulance) can be learned without hands-on training successfully. Young children should be able mainly to recognize the emergency situation and call the ambulance. In our study, the participants were able to call the ambulance in a high proportion (> 90% when using the teaching cards).

Despite the promising results, there is a need for further studies in this area to develop an evidence-based background and reach as many children successfully as possible. Furthermore, based on the present results young schoolchildren are able to learn the different elements of the BLS sequence separately, but it is difficult to carry out the whole process correctly and in the appropriate order. For this reason, training content should strongly adapt to the psychomotor development of children. Teaching cards could be useful for 6–10-year old schoolchildren to introduce the importance of giving help, however, combined with hands-on training could also be useful.

### Limitations

Our study has several limitations. First, it might not be representative of the whole population of primary school children in Hungary (or all over the world). Sociodemographic differences between the schools could affect results, however, randomization based on the three different schools made it possible to avoid contaminating the different methods in the same school. On the other hand, randomization based on the schools could cause other biases (differences in characteristics of the group members). Second, we do not have information about the usage of teaching cards as self-instruction kits. Third, differences between the teaching times in the different groups (45 min vs. 15 min) could also affect the results, however, the time of the theoretical background was the same in every group (15 min). Training with other instructors (e.g. teachers) could lead to other results. In addition, children were re-tested after two months of training, but we do not know the longer-lasting effects of training. Furthermore, we found some baseline differences between the groups. Despite our assumption, we do not know the exact reason for the differences between the teaching card and combined teaching groups. Finally, the actual reaction of these children in a possible real emergency situation remains unknown.

## Conclusion

Using alternative and low-cost materials for teaching BLS to young schoolchildren can be effective. Teaching cards were not inferior compared to traditional and combined methods in some skills but inferior in others. Therefore, hands-on training opportunity is still important. However, good-quality and long-term skill performance were observed in the group using only teaching cards after two months. Attitude did not change over time and was the lowest in the teaching card group. To learn correctly the whole sequence of BLS is difficult for 6 to 10 years-old children, however, they are able to learn more BLS-related skills separately. Low-cost methods may hold the potential to reach a larger target population, implement BLS into the school curricula and support the KIDS SAVE LIVES project but further studies are needed.

## Data Availability

The datasets used and/or analysed during the current study available from the corresponding author on reasonable request.

## Abbreviations

AED: Automated External Defibrillator
BLS: Basic Life Support
CPR: Cardiopulmonary resuscitation
ERC: European Resuscitation Council
OHCA: Out-of-hospital cardiac arrest
CG: Combined group
TG: Traditional group
TCG: Teaching card group
T0: Pre-test (before the training)
T1: Test after the training (within one week)
T2: Test after two months

## References

[1] Gräsner JT, Herlitz J, Tjelmeland IBM (2021). European resuscitation council guidelines 2021: epidemiology of cardiac arrest in europe. Resusc.

[2] Malta Hansen C, Kragholm K, Pearson DA (2015). Association of bystander and first-responder intervention with survival after out-of-hospital cardiac arrest in North Carolina, 2010–2013. JAMA.

[3] Wissenberg M, Lippert FK, Folke F (2013). Association of national initiatives to improve cardiac arrest management with rates of bystander intervention and patient survival after out-of-hospital cardiac arrest. JAMA.

[4] Coons SJ, Guy MC (2009). Performing bystander CPR for sudden cardiac arrest: behavioral intentions among the general adult population in Arizona. Resusc.

[5] Gräsner JT, Lefering R, Koster RW (2016). EuReCa ONE – 27 Nations, ONE Erurope, ONE Registry. A prospective one months analysis of out-of-hospital cardiac arrest outcomes in 27 contries in Europe. Resusc.

[6] Greif R, Lockey A, Breckwoldt J (2021). European resuscitation council guidelines 2021: education for resuscitation. Resusc.

[7] Semeraro F, Greif R, Böttiger BW (2021). European resuscitation council guidelines 2021: systems saving lives. Resusc.

[8] Scapigliati A, Zace D, Matsuyama T (2021). Community initiatives to promote basic life support implementation – a scoping review. J Clin Med.

[9] Nakagava NK, Salles IC, Semeraro F (2021). KIDS SAVE LIVES: a narrative review of associated scientific production. Curr Op Crit Care.

[10] Nakagawa NK, Silva LM, Carvalho-Oliveira R (2019). KIDS SAVE LIVES BRAZIL: A succesful pilot program to implement CPR at primary and high school in Brazil resulting in a state law for a training CPR week. Resusc.

[11] Bánfai B, Bánfai-Csonka H, Musch J (2021). KIDS SAVE LIVES in Hungary (KSLH): Overview of the last two years-How does it work and how could it be better with children and teachers?. Resusc.

[12] Böttiger BW, Bossaert LL, Castrén M, et al. Kids Save Lives – ERC Position statement on schoolchildren education in CPR: “Hands that help – Training children is training for life.” Resusc. 2016;105:A1–3. 10.1016/j.resuscitation.2016.06.005 27339096

[13] Böttiger BW, Lockey A, Georgiou M (2020). KIDS SAVE LIVES: ERC Position statement on schoolteachers’ education and qualification in resuscitation. Resusc.

[14] Mpotos N, Vekeman E, Monsieurs K (2013). Knowledge and willingness to teach cardiopulmonary resuscitation: A survey amongst 4273 teachers. Resusc.

[15] Hansen CM, Zinckernagel L, Ersboll AK (2017). Cardiopulmonary resuscitation training in schools following 8 years of mandating legislation in Denmark: A nationwide survey. J Am Heart Assoc.

[16] Abelairas-Gómez C, Schroeder DC, Carballo-Fazanes A (2021). KIDS SAVE LIVES in schools: cross-sectional survey of schoolteachers. Eur J Pediatr.

[17] Liberman M, Golberg N, Mulder D (2000). Teaching cardiopulmonary resuscitation to CEGEP students in Quebec–a pilot project. Resusc.

[18] Kaye W, Rallis SF, Mancini ME (1991). The problem of poor retention of cardiopulmonary resuscitation skills may lie with the instructor, not the learner or the curriculum. Resusc.

[19] Lau Y, Nyoe RSS, Wong SN (2018). Effectiveness of digital resuscitation training in improving knowledge and skills: a systematic review and meta-analysis of randomised controlled trials. Resusc.

[20] Varela-Casal C, Abelairas-Gomez C, Otero-Agra M (2021). Teaching Basic Life Support to 5- to 8-year-old children: A cluster randomized trial. Pediatrics.

[21] Bánfai B, Pandur A, Schiszler B (2019). The (second) year of first aid’: a 15-month follow-up after a 3-day first aid programme. Emerg Med J.

[22] Bánfai B, Pandur A, Schiszler B (2018). Little lifesavers: Can we start first aid education in kindergarten? – A longitudinal cohort study. H Educ J.

[23] Plant N, Taylor K (2013). How best to teach CPR to schoolchildren: a systematic review. Resusc.

[24] De Buck E, Van Remoortel H, Dieltjens T (2015). Evidence-based educational pathway for the integration of first aid training in school curricula. Resuscitation..

[25] Perkins GD, Handley AJ, Koster RW (2015). Adult Basic Life Support and Automated External Defibrillation Section Collaborators. European Resuscitation Council guidelines for resuscitation 2015: Section 2. Adult basic life support and automated external defibrillation. Resuscitation.

[26] Ko YC, Yang CW, Lin HY (2021). A non-inferiority randomised controlled trial comparing self-instruction with instructor-led method in training of layperson cardiopulmonary resuscitation. Sci Rep.

[27] Böttiger BW, Van Aken H (2015). Kids save lives – Training school children in cardiopulmonary resuscitation worldwide is now endorsed by the World Health Organisation (WHO). Resusc.

[28] Jones I, Whitfield R, Colquhoun M (2007). At what age can schoolchildren provide effective chest compressions? An observational study from the Heartstart UK schools training programme. BMJ.

[29] Abelairas-Gómez C, Rodriguez-Núnez A, Casillas-Cabana M (2014). Schoolchildren as life savers: At what age do they become strong enough?. Resusc.

[30] Berthelot S, Plourde M, Bertrand I (2013). Push hard, push fast: quasy experimental study on the capacity of elementary schoolchildren to perform cardiopulmonary resuscitation. Scand J Trauma Resusc Emerg Med.

[31] Bollig G, Myklebust AG, Ostringen K (2011). Effects of first aid training in the kindergarten – a pilot study. Scand J Trauma Resusc Emerg Med.

[32] Nas J, Thannhauser J, Vart P (2020). Effect of face-to-face vs Virtual Reality training on cardiopulmonary resuscitation quality. A Randomized Clinical Trial JAMA Cardiol.

[33] Issleib M, Kromer A, Pinnschmidt HO (2021). Virtual reality as a teaching method for resuscitation training in undergraduate first year medical students: a randomized controlled trial. Scand J Trauma Resusc Emerg Med.

[34] Hsieh MJ, Bhanji F, Chiang WC (2016). Comparing the effect of self-instruction with that traditional instruction in basic life support courses – A systematic review. Resusc.

